# Mining published lists of cancer related microarray experiments: Identification of a gene expression signature having a critical role in cell-cycle control

**DOI:** 10.1186/1471-2105-6-S4-S14

**Published:** 2005-12-01

**Authors:** Giacomo Finocchiaro, Francesco Mancuso, Heiko Muller

**Affiliations:** 1 European Institute of Oncology, Via Ripamonti 435, 20141 Milan, Italy; 2 IFOM Foundation, Via Adamello 16, 20139 Milan, Italy

## Abstract

**Background:**

Routine application of gene expression microarray technology is rapidly producing large amounts of data that necessitate new approaches of analysis. The analysis of a specific microarray experiment profits enormously from cross-comparing to other experiments. This process is generally performed by numerical meta-analysis of published data where the researcher chooses the datasets to be analyzed based on assumptions about the biological relations of published datasets to his own data, thus severely limiting the possibility of finding surprising connections. Here we propose using a repository of published gene lists for the identification of interesting datasets to be subjected to more detailed numerical analysis.

**Results:**

We have compiled lists of genes that have been reported as differentially regulated in cancer related microarray studies. We searched these gene lists for statistically significant overlaps with lists of genes regulated by the tumor suppressors p16 and pRB. We identified a highly significant overlap of p16 and pRB target genes with genes regulated by the EWS/FLI fusion protein. Detailed numerical analysis of these data identified two sets of genes with clearly distinct roles in the G1/S and the G2/M phases of the cell cycle, as measured by enrichment of Gene Ontology categories.

**Conclusion:**

We show that mining of published gene lists in the absence of numerical detail about gene expression levels constitutes a fast, easy to perform, widely applicable, and unbiased route towards the identification of biologically related gene expression microarray datasets.

## Background

Recent technological advances have profoundly changed the nature of biological research in general and of cancer research in particular. Work in the previous years has unveiled the building blocks of life (genes) in more than 100 different organisms, including humans [1]. High throughput technologies have been developed that allow the measurement of gene expression, protein interactions, and SNPs on a genome wide scale and to correlate such data with disease. The challenge now is to turn the enormous amount of data into better understanding and, eventually, therapies for cancer and other human diseases.

Since the introduction of high throughput gene expression screening into biological research, pioneered by the laboratory of P.O. Brown [2] a decade ago, a tremendous amount of data has been accumulated. Several microarray projects have generated large compendia of gene expression data that provide a comprehensive view of the transcriptome in various organisms at different stages of development as well as in different environmental or genetic conditions [3-5]. Public repositories have been developed that host a significant amount of published data, although the coverage is far from complete [6,7].

The prevailing use of high throughput gene expression screening tools focuses on a restricted set of biological conditions and genome wide expression profiles for these conditions are being generated. Once the data have been analyzed statistically and validated for a number of genes, the interpretation of the data constitutes the main bottleneck towards the identification of biologically meaningful results. Meta-analysis methods have been devised that can help the biologist interpreting the data in the context of other gene expression data sets [8-10]. Nevertheless, researchers often limit their meta-analysis efforts to a small number of data sets that report results on the study of the same or similar biological systems. The raw data are generally downloaded from the web and numerical data analysis of the published and in-house generated data is performed in parallel. Obviously, in the light of the ever growing number of published datasets, this analysis mode quickly meets its limits. Furthermore, the hypothesis driven way of choosing published datasets for meta-analysis constitutes a servere limitation towards identifying unexpected connections between dissimilar datasets. Currently, there is no resource available that helps the biologists in the identification of datasets that report genes similar to the ones he or she is interested in.

We have explored the feasibility of mining published lists of regulated genes for the identification of published microarray datasets to be used in meta-analysis. Specifically, we stored lists of regulated genes derived from more than 150 publications. The repository of gene lists was searched using p16 and pRB target genes [11]. We find a highly significant overlap of these lists with genes regulated by the EWS/FLI fusion protein [12], which is detected in more than 95% of Ewing's sarcoma family of tumors [13]. By cluster analysis of the the raw data, we extracted two signatures differentially regulated by p16, pRB, and EWS/FLI. These signatures display clearly distinct patterns of enrichment of Gene Ontology categories. One cluster contains genes whose function is specific to G1/S and the other cluster contains genes whose function is specific to the G2/M phases of the cell cycle. These results suggest that mining published lists of regulated genes provides a convenient, fast, and unbiased way for identifying biologically related datasets.

## Methods

### Generation of a repository of published lists of cancer related microarray experiments

We queried the Affymetrix database of scientific publications. The database contains more than 3000 scientific publications that use or review GeneChip^® ^technology. We selected 155 papers concerning both expression profiling of cancer specimens and mechanistic studies of cancer related genes.

Medline annotations of these papers were downloaded using a perl script calling NCBI Entrez Utilities  via the LWP module. XML files were parsed by a perl script using the DOM module.

Gene lists were extracted manually from publications and were annotated using a procedure similar to the one used to generate IFOM DNA chip annotations tables [14]. Data regarding publications and published gene lists were relationally linked in a MySQL database.

### Analysis of p16, pRB microarray data

Data analysis was performed using GenePicker [15]. GenePicker allows the user to set up analysis schemes and to search the data for regulated genes using t-test, ANOVA and Change-FoldChange analysis. Genes passing t-test and Change-FoldChange analysis were selected. Near optimal analysis parameters were defined using a genetic algorithm implemented in GenePicker.

### Identification of published lists enriched in p16/pRB regulated genes

The overlap between p16 and pRB lists of regulated genes and the lists in the repository was evaluated by determining the number of common NCBI Entrez Gene identifiers between the annotated lists in the repository and the lists of p16/pRB regulated genes. For this process to work efficiently, each microarray platform in the repository was annotated with corresponding NCBI Entrez Gene identifiers and each published list was associated to the microarray platforms indicated in the corresponding publication. The significance of overlap of lists of genes regulated by pRB or p16 and gene lists annotated in the repository can be assessed using a sampling without replacement model. The corresponding P-value is calculated using the hypergeometric distribution:



where *k *represents the number of common NCBI Entrez Gene identifiers, *n *represents the corrected size of pRB/p16 lists after the elimination of NCBI Entrez Gene identifiers present in the list of p16/pRB regulated genes but not present on the reference platform. *N *is the number of NCBI Entrez Gene identifiers present on the reference platform, *K *is the number of annotated Gene identifiers in the published list that is being analyzed. We applied Benjamini-Hochberg false discovery rate as multiple testing correction [16].

### Clustering method to expand signatures

Given the two different signatures extracted by mining our repository we identified other genes with similar behavior in pRB, p16, and EWS/FLI raw datasets. We extracted raw values for each probeset included in the original signature. This represented our initial cluster of genes and then we calculated a median centroid representing the genes of this cluster separately for the pRB, p16, and EWS/FLI dataset. In each class (pRB, p16, and EWS/FLI), we calculated the Pearson correlation coefficient r, between every gene and the representative centroid. The correlation coefficient r was calculated separately for each class for the weight of each class to be independent of the number of samples in the class. This analysis resulted in three correlation coefficients for every gene, one for each class. Genes with an average correlation coefficient  greater than 0.8 were selected for further analysis.  is defined by



where N is the number of classes (3) and r_i _is the Pearson correlation coefficient of the analyzed gene in the class *i*.

We evaluated the quality of expanded clusters of size n in each class as follows: (i) we calculated, for each gene in the cluster, the Pearson correlation coefficient with other genes in the expanded cluster separately for each class of samples pRB, p16, and EWS/FLI; (ii) we selected the n(n-1)/2 non redundant values of this correlation matrix; (iii) the average Acv (Average of correlation values) and standard deviation of these values is considered as an indicator of similarity of the gene expression profiles of the genes composing the cluster.

## Results

### Details on extracted signatures

Publications on cancer related microarray experiments were extracted from the Affymetrix database of scientific publications . We retrieved 155 papers reporting both expression profiling of cancer specimens and experiments on genes that have been identified as being involved in oncogenesis. Medical Subject Headings of class G (biological sciences) like Cell Division, Signal Transduction, Cell Differentiation, Gene Amplification and Chromosome Deletion are strongly represented in the set of publications we considered. Some publications contain multiple gene lists classified with different criteria. Thus, altogether we stored and annotated 708 gene lists in our repository.

Gene identifiers were annotated using a procedure similar to the one used to generate IFOM DNA chip annotation tables [14]. In total, we identified 7225 Entrez Gene identifiers, representing about the 22% of the Homo sapiens records in Entrez Gene and 35% of NCBI Entrez Gene identifiers detectable by Affymetrix microarrays.

### Microarray Experiments on pRB and p16

Two in-house generated gene lists derived from experiments on cell lines that conditionally express either a constitutively active mutant of pRB or p16INK4A [11] were used to search the repository. The HG-U95A subset of the p16 and pRB microarray data was analyzed using GenePicker [15] (see Methods). We identified 200 genes down regulated and 128 up regulated by pRB as well as 126 genes down regulated and 19 up regulated by p16 (Additional file 1).

### Identification of published signatures having a significant overlap with p16 and pRB regulated genes

**Figure 1:**
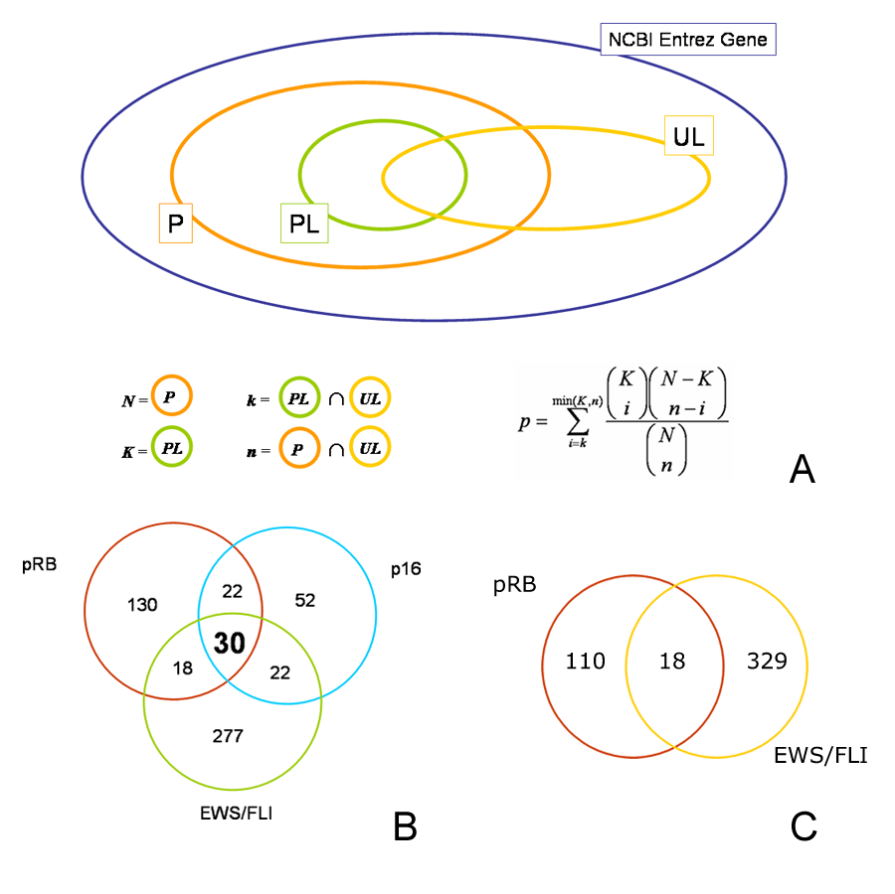
**Identification of published lists enriched in p16/pRB regulated genes**. **(A) **Each circle in the figure represents a set of NCBI Gene identifiers (whole set, blue circle). The significance of the amount of NCBI Gene identifiers in common (***k***) between p16 or pRB regulated genes (UL, yellow circle) and one published list (PL, green circle) is evaluated using the hypergeometric distribution (the formula is reported in right bottom corner of panel A). For each platform (P, orange circle) we annotated the subset of NCBI Gene identifiers that are present on the platform used in the published work (***N***). The significance of the overlap is estimated considering only NCBI Gene identifiers present in the user list and the P platform (***n***). **(B) **Venn diagram representing the overlap of NCBI Gene identifiers repressed by pRB, p16, and EWS/FLI **(C) **Venn diagram representing the overlap of NCBI Gene identifiers induced by pRB and repressed by EWS/FLI.

We searched for gene lists significantly overlapping with genes regulated by p16 and pRB by calculating the corresponding P-value according to the hypergeometric distribution (Fig. 1A and Methods section). The overlap between lists was estimated based on common NCBI Entrez Gene identifiers. The results are reported in Table 1 (p-value FDR corrected < 0.05). As an indication of the reliability of our approach, gene lists with the most significant overlap reported genes regulated by pRB, p16 and E2F [11,17]. Interestingly, however, the genes regulated by p16 and pRB were highly enriched for genes regulated by the EWS/FLI fusion protein in primary human fibroblasts [12]. This chimeric protein is generated by the chromosomal translocation t(11;22)(q24q12) that is detected in more than 95% of Ewing's sarcoma family of tumors [13]. The fusion protein facilitates tumorigenesis but further mutations are required [12]. Among the three lists reported by [12], the strongest over-representation of pRB and p16 regulated genes was detected for the list of genes down-regulated by EWS/FLI. Specifically, 30 genes are downregulated by pRB, p16 and EWS/FLI (p-value < 1e-6, Figure 1B), whereas 18 genes are downregulated by EWS/FLI but upregulated by pRB (p-value = 0.0007, Figure 1C).

### Expansion of the signatures

The illustrated approach allowed us to identify common targets of pRB, p16 and EWS/FLI having an annotated NCBI Entrez Gene identifier. However, the p16-pRB data and the EWS/FLI data were analyzed using different methods. Therefore, we asked whether other genes – not annotated with a NCBI Entrez Gene identifier or not extracted by a particular type of analysis – behave similarly to the identified common target genes. This analysis was performed using the raw data. The EWS/FLI dataset was downloaded from  and combined with the p16-pRB data. All the data were median centered per array and probesets that did not detect expression of the corresponding gene in at least two samples per class according to Affymetrix MAS5 Present call [18] were eliminated. The overlapping genes identified previously were represented by 39 Affymetrix probesets corresponding to 30 genes repressed by pRB, p16, EWS/FLI, and 21 probesets representing 18 genes repressed by EWS/FLI but induced by pRB (Additional files 1 and 2).

**Figure 2:**
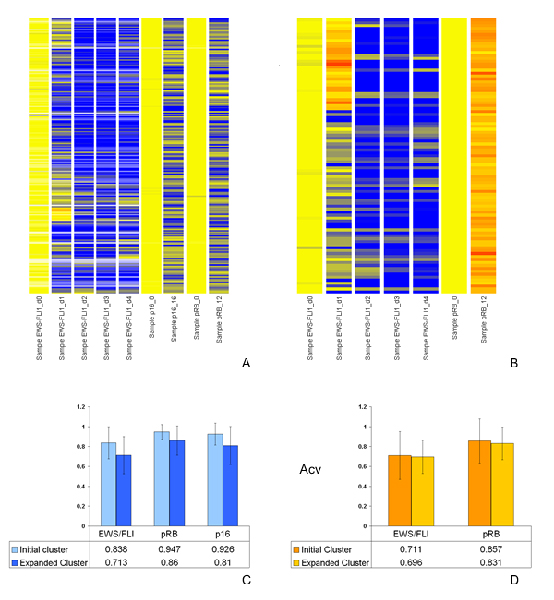
**Expanded signatures**. **(A) **210 probesets down regulated by EWS/FLI, p16, and pRB. **Sample EWS-FLI1_d0**, sample of EWS/FLI dataset analyzed at day 0; **Sample p16_0**, sample of p16 dataset analyzed at 0 hours; **Sample pRB_0**, sample of pRB dataset analyzed at 0 hours. Red means up regulation, relative to control conditions (Sample EWS-FLI1_d0 for EWS/FLI dataset; Sample p16_0 for p16 dataset; Sample pRB_0 for pRB dataset), blue means down regulation. **(B) **93 probesets up regulated by pRB and down regulated by EWS/FLI. **(C) **Average of correlation values (Acv) for initial set of probesets identified mining published lists (initial cluster) and for the expanded cluster. Acv are calculated on probesets down regulated by EWS/FLI, pRB, and p16. Error bars represent the standard deviation of the correlation values. **(D) **Acv calculated for probesets up regulated by pRB and down regulated by EWS/FLI.

Both signatures were expanded by cluster analysis (see Methods section for details): Briefly, genes that are strongly correlated with the genes of the original cluster were identified separately in each data set (pRB, p16, EWS/FLI) by calculating the Pearson correlation coefficient of each gene to the median centroid of the original genes. The expanded cluster was formed by including genes whose mean correlation coefficient in the three classes was superior to 0.8. This analysis allowed us to expand the initial cluster to 210 probesets down regulated by pRB, p16 and EWS/FLI and 93 probesets up regulated by pRB but down regulated by EWS/FLI. The results are depicted in Figure 2. In order to evaluate the quality of the expanded cluster, we calculated the average correlation coefficient between all genes of the expanded cluster (separately within each class of samples) and compared it to the average correlation coefficient observed between genes of the original cluster (see Methods section for details on calculating the average correlation coefficient, Acv). This analysis indicated that the average correlation of the expanded gene set is very similar to the average correlation observed in the initial gene set. Acv values and the corresponding standard deviations are reported in Figure 2C and 2D.

The analysis on Affymetrix raw data was performed using MAS5 [18]. Alternative techniques of data normalization are available. In order to test how the normalization procedure affects the results of our analysis, EWS/FLI, pRB, and p16 datasets were independently processed with RMA [19] and GCRMA [20]. Values of the probesets composing the two expanded signatures were determined and average correlation value analysis was performed as described for MAS5 data. We did not observe a dramatic variation of the distribution of correlation values using different techniques of normalization (Additional file 2).

### Functional classification of expanded signatures

The characterization of the two expanded signatures was performed by evaluating the enrichment of Gene Ontology categories. The analysis was conducted with GoTM [21] and two different types of reference gene sets were used. First, we considered as a reference set the genes that are detectable by Affymetrix HG-U95Av2 platform to have a global overview of biological processes, molecular functions and cellular localizations of genes in the expanded signatures. Second, to evaluate the relative enrichment of one signature for a particular Gene Ontology category, we used the merger of the two expanded signatures as a reference set.

**Figure 3:**
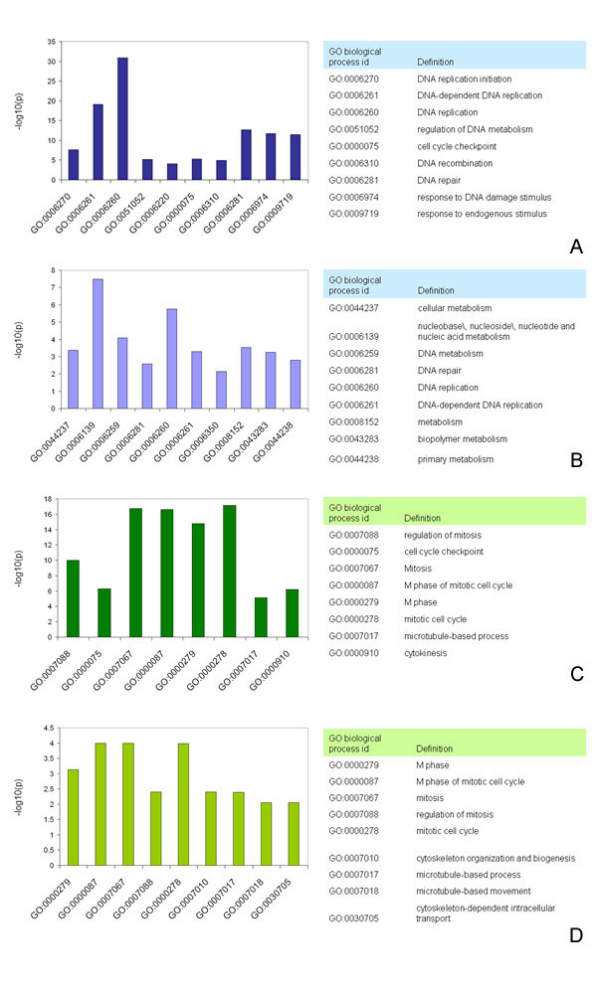
**Enrichment in Gene Ontology categories**. The x axis of the histogram reports the identifier of the Gene Ontology category, the y axis reports -log_10 _P, where P represents the significance of the enrichment evaluated by GoTM. **(A) **Analysis of expanded cluster of genes repressed by pRB, p16, and EWS/FLI using, as reference gene list, NCBI Gene identifiers annotated in the Affymetrix HG_U95Av2 platform. Plotted categories show enrichment greater than 5 fold and occur more than 5 times in the expanded cluster. **(B) **Analysis of expanded cluster of genes repressed by pRB, p16, and EWS/FLI using as reference gene list the two expanded clusters merged. All the significantly enriched categories are plotted **(C) **Analysis of expanded cluster of genes induced by pRB, and repressed by EWS/FLI using as reference gene list, NCBI Gene identifiers annotated in Affymetrix HG_U95Av2 platform. Only categories enriched more than 5 fold and occuring more than 5 times are represented. **(D) **Analysis of expanded cluster of genes induced by pRB, and repressed by EWS/FLI using as reference gene list the merger of the two expanded clusters.

The results of enriched GO biological process categories are shown in Figure 3. We found that both expanded sets are strongly enriched for genes involved in cell cycle regulation. However, they seem to play a role in different phases of the cell cycle. Genes repressed by pRB, p16 and EWS/FLI are mainly involved in processes like DNA replication (*CCNH*, *EXO1*, *PCNA*, *FANCL*, *RAD54L*) and DNA repair (*POLA*, *SSBP1*, *CDC45L*, *RFC5*). Among the genes that are induced by pRB but repressed by EWS/FLI, the predominant group of genes are active during M phase, like *BUB1 *and *BUB3 *(involved in the mitotic spindle checkpoint) and *CCNB1*, *CCNB2*, and *CDC25C *that regulate progression through mitosis.

## Conclusion

Gene expression screenings have become routine in many laboratories and the Affymetrix database of published articles using their technology counts more then 3000 citations where Affymetrix technology is only one out of several. Public repositories have been established [6,7] (ArrayExpress and Gene Expression Omnibus (GEO)) that, however, still host only a minor portion of published gene expression data (373 experiments in ArrayExpress and 639 datasets in GEO containing also SAGE data as of October 2004). Furthermore, due to the need for technological detail in the database entries, a simple query like "How is my gene behaving in the published datasets?" cannot be carried out easily even though this type of query is needed for efficient meta-analysis of gene expression data. The question that is being addressed by most gene expression screenings is: "What genes change expression in a specific condition?" In order to perform efficient meta-analysis of gene expression data the question to be answered is: "What conditions make this gene change expression?"

In order to facilitate answering this question, we set up a dedicated database where researchers can find and query lists of genes that have been reported in published microarray screenings. Two basic types of information are stored in this database: What genes have been interrogated in a given experiment (array platform) and what genes have been found regulated or behave as classifiers of tumor samples. Technical details and numerical hybridization results are not included. The main use of the database is to find published reports on genes of interest. In order for this type of query to work efficiently, high quality annotations of the reported gene lists is necessary to enable unequivocal gene identification across experiments. At IFOM, we have currently established such an annotation pipeline that is automated and satisfies this need [14]. The user has access to Pubmed abstracts and all gene lists reported in a particular paper.

To illustrate the utility of this resource, here we demonstrate that by searching the database with lists of genes that are regulated by p16 and pRB in cellular model systems, an unexpectedly strong overlap with genes regulated by the EWS/FLI fusion protein can be detected. The set of genes that is regulated by p16 and pRB on the one hand and genes reported to be regulated by EWS/FLI on the other hand has been used as a seed cluster that was expanded by detailed numerical analysis of the raw data. Analysis of the genes in the expanded cluster for the enrichment of Gene Ontology categories reveals that most genes are involved in the regulation of the cell cycle. However, subcategories can be identified. Specifically, the list of genes that are down regulated by pRB, p16, and EWS/FLI are strongly enriched for genes that function in DNA replication and DNA repair whereas genes that are up regulated by pRB and down regulated by EWS/FLI are enriched for genes with mitotic functions. Although pRB and p16 are best known for their role in the regulation of the G1/S transition, several reports have identified genes with a role in G2/M that are under the control of pRB/p16 as E2F target genes [22-24]. Thus, it seems likely that the strong enrichment for genes with functions in G1/S and G2/M in the set of genes that are regulated by both pRB/p16 and EWS/FLI reflect physiological mechanisms of gene expression control. It is tempting to speculate that EWS/FLI subverts the expression of pRB/p16 target genes by an unknown mechanism and that this event facilitates tumorigenesis that, however, requires additional mutations [12]. If this mechanisms applies, it is likely that pRB/p16 function is only partially compromised by EWS/FLI because Ewing's Sarcomas with a deletion of p16INK4A are characterized by a more aggressive behaviour and poorer response to chemotherapy than Ewing's sarcomas with functional p16 [25]. The significance of the signatures identified in this study remains to be validated by experimental means. However, the identification of common targets of pRB/p16 and EWS/FLI pathways reported here may help to elucidate the molecular mechanisms leading to the development of Ewing's sarcoma.

## List of abbreviations used

Acv: Average of correlation values

## Supplementary data



## Supplementary Material

Additional File 1 This file contains lists of regulated genes. The initial analysis of p16, pRB expermients is reported in the worksheets pRB regulated, p16 regulated. Overlapping probesets extracted by mining published lists are indicated in worksheets: 'p16, pRB, EWS-FLI down start clus', 'EWS-FLI down, pRB up start clus'. The worksheets 'p16, pRB, EWS-FLI down expan clus', 'EWS-FLI down, pRB up expan clus' contain the expanded lists. Click here for file

Additional File 2 Details on cluster generation are reported: the average of correlation values and the related standard deviations are shown. Moreover, the distribution of correlation values is illustrated in histograms. Click here for file

## Figures and Tables

**Table 1:** Analysis of published lists. Publications reporting regulated genes that are highly over-represented (P-value FDR corrected < 0.05) in p16 regulated genes (Table 1A) and pRB regulated genes (Table 1B).

**A**				
**Pubmed ID**	**Authors**	**Year**	**Reference**	**P-value**

12086853	Lessnick, S. L., et al.	2002	Cancer Cell 1(4): 393–401.	3.56E-27
12923195	Vernell, R., et al.	2003	J Biol Chem 278(46): 46124–37.	7.71E-24
12874028	Barrett, M. T., et al.	2003	Cancer Res 63(14): 4211–7.	7.59E-17
12782787	Rozovskaia, T., et al.	2003	Proc Natl Acad Sci U S A 100(13): 7853–8.	1.18E-08
12154061	LaTulippe, E., et al.	2002	Cancer Res 62(15): 4499–506.	1.38E-08
11929952	Agrawal, D., et al.	2002	J Natl Cancer Inst 94(7): 513–21.	0.000006
12819026	Hansel, D. E., et al.	2003	Am J Pathol 163(1): 217–29.	0.00003
12637319	Ferrando, A. A., et al.	2003	Blood 102(1): 262–8.	0.00008
14578194	Borczuk, A. C., et al.	2003	Am J Pathol 163(5): 1949–60.	0.000992
14562049	Young, A. P.,, et al.	2003	Oncogene 22(46): 7209–17.	0.001446
12791645	Croonquist, P. A., et al.	2003	Blood 102(7): 2581–92.	0.001758
14522919	Lapillonne, H., et al.	2003	Cancer Res 63(18): 5926–39.	0.008576
11181568	Lawrance, I. C., et al.	2001	Hum Mol Genet 10(5): 445–56.	0.012072
11267935	Akiyoshi, S., et al.	2001	Jpn J Cancer Res 92(3): 257–68.	0.012383
11559565	Rickman, D. S., et al.	2001	Cancer Res 61(18): 6885–91.	0.022683
12460888	Bar-Shira, A., J. et al.	2002	Cancer Res 62(23): 6803–7.	0.036384
12629520	Presneau, N., et al.	2003	Oncogene 22(10): 1568–79.	0.036956
12468598	Li, Y. and F. H. Sarkar	2002	J Nutr 132(12): 3623–31.	0.046238

**B**				

**Pubmed ID**	**Authors**	**Year**	**Reference**	**P-value**

12086853	Lessnick, S. L., et al.	2002	Cancer Cell 1(4): 393–401.	7.39E-21
12923195	Vernell, R., et al.	2003	J Biol Chem 278(46): 46124–37.	1.92E-18
12874028	Barrett, M. T., et al.	2003	Cancer Res 63(14): 4211–7.	8.23E-15
11929952	Agrawal, D., et al.	2002	J Natl Cancer Inst 94(7): 513–21.	8.37E-08
12468598	Li, Y. and F. H. Sarkar	2002	J Nutr 132(12): 3623–31.	0.000001
12154061	LaTulippe, et al.	2002	Cancer Res 62(15): 4499–506.	0.000006
14522919	Lapillonne, et al.	2003	Cancer Res 63(18): 5926–39.	0.000021
12819026	Hansel, D. E., et al.	2003	Am J Pathol 163(1): 217–29.	0.000066
12198119	Gajate, C., et al.	2002	J Biol Chem 277(44): 41580–9.	0.000074
14578194	Borczuk, A. C., et al.	2003	Am J Pathol 163(5): 1949–60.	0.000092
12782787	Rozovskaia, T., et al.	2003	Proc Natl Acad Sci U S A 100(13): 7853–8.	0.001664
14562049	Young, A. P., et al.	2003	Oncogene 22(46): 7209–17.	0.002554
11267935	Akiyoshi, S., et al.	2001	Jpn J Cancer Res 92(3): 257–68.	0.003859
11966535	Sepulveda, A. R., et al.	2002	Aliment Pharmacol Ther 16 Suppl 2: 145–57.	0.013999
11559565	Rickman, D. S., et al.	2001	Cancer Res 61(18): 6885–91.	0.014114
12068005	Pise-Masison, et al.	2002	Cancer Res 62(12): 3562–71.	0.019419
12791645	Croonquist, P. A., et al.	2003	Blood 102(7): 2581–92.	0.024727
12637319	Ferrando, A. A., et al.	2003	Blood 102(1): 262–8.	0.02533
