# Supplementary material for: Mining published lists of cancer related microarray experiments: Identification of a gene expression signature having a critical role in cell-cycle control
Source: BMC Bioinformatics. 2005 Dec 1;6(Suppl 4):S14. doi: 10.1186/1471-2105-6-S4-S14 (PMC1866394; doi:10.1186/1471-2105-6-S4-S14)
Supplement: Additional File 2 — Details on cluster generation are reported: the average of correlation values and the related standard deviations are shown. Moreover, the distribution of correlation values is illustrated in histograms. [file 1471-2105-6-S4-S14-S2.doc]

| |  | | --- | |
| --- | --- |

|  |  | Mining published lists of cancer related microarray experiments: Identification of a gene expression signature having a critical role in cell-cycle control  **Details on expanded clusters**  **Average of correlation values**   |  | **EWS/FLI** | **pRB** | **p16** | | --- | --- | --- | --- | | **p16, pRB, EWS/FLI downregulated probesets, initial set** | 0.711 | 0.857 |  | | **p16, pRB, EWS/FLI downregulated probesets, expanded set** | 0.696 | 0.831 |  | | **EWS/FLI downregulated, pRB upregulated initial cluster** | 0.838 | 0.947 | 0.926 | | **EWS/FLI downregulated, pRB upregulated expanded cluster** | 0.713 | 0.86 | 0.81 |     **Standard deviation of correlation values**   |  | **EWS/FLI** | **pRB** | **p16** | | --- | --- | --- | --- | | **p16, pRB, EWS/FLI downregulated probesets, initial set** | 0.241 | 0.222 |  | | **p16, pRB, EWS/FLI downregulated probesets, expanded set** | 0.168 | 0.164 |  | | **EWS/FLI downregulated, pRB upregulated initial set** | 0.161 | 0.074 | 0.111 | | **EWS/FLI downregulated, pRB upregulated expanded set** | 0.187 | 0.147 | 0.191 |     **Distribution of correlation values**   | **EWS/FLI downregulated, pRB upregulated expanded set**   | Mas5 | GCRMA | RMA | | --- | --- | --- | | [EWS-FLI](../images/MAS5/pRB_up_EWS_MAS5.jpg) | [EWS-FLI](../images/GCRMA/pRB_up_EWS_GCRMA.jpeg) | [EWS-FLI](../images/RMA/pRB_up_EWS_RMA.jpeg) | | [pRB](../images/MAS5/pRB_up_pRB_MAS5.jpg) | [pRB](../images/GCRMA/pRB_up_pRB_GCRMA.jpeg) | [pRB](../images/RMA/pRB_up_pRB_RMA.jpeg) | | | --- | --- | --- | --- | --- | --- | --- | --- | --- | --- | | **p16, pRB, EWS/FLI downregulated expanded set**   | Mas5 | GCRMA | RMA | | --- | --- | --- | | [EWS-FLI](../images/MAS5/all_down_EWS_MAS5.jpg) | [EWS-FLI](../images/GCRMA/all_down_EWS_GCRMA.jpeg) | [EWS-FLI](../images/RMA/all_down_EWS_RMA.jpeg) | | [pRB](../images/MAS5/all_down_pRB_MAS5.jpg)  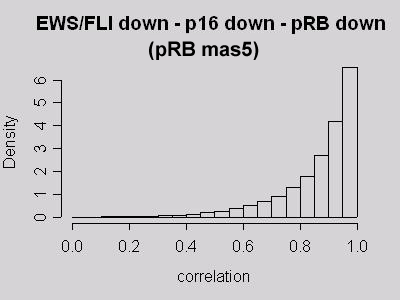 | [pRB](../images/GCRMA/all_down_pRB_GCRMA.jpeg) | [pRB](../images/RMA/all_down_pRB_RMA.jpeg) | | [p16](../images/MAS5/all_down_p16_MAS5.jpg)  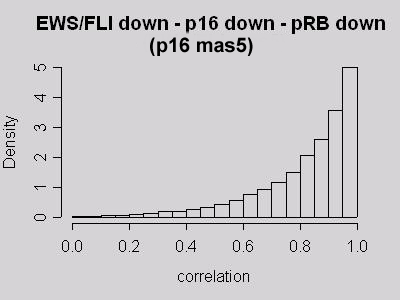 | [p16](../images/GCRMA/all_down_p16_GCRMA.jpeg) | [p16](../images/RMA/all_down_p16_RMA.jpeg) | | |  |
| --- | --- | --- | --- | --- | --- | --- | --- | --- | --- | --- | --- | --- | --- | --- | --- | --- | --- | --- | --- | --- | --- | --- | --- | --- | --- | --- | --- | --- | --- | --- | --- | --- | --- | --- | --- | --- | --- | --- | --- | --- | --- | --- | --- | --- | --- | --- | --- | --- | --- | --- | --- | --- | --- | --- | --- | --- | --- | --- | --- | --- | --- | --- | --- | --- | --- | --- |
